# Concurrent radiochemotherapy in locally-regionally advanced oropharyngeal squamous cell carcinoma: analysis of treatment results and prognostic factors

**DOI:** 10.1186/1748-717X-7-78

**Published:** 2012-05-28

**Authors:** Valentina Krstevska, Igor Stojkovski, Beti Zafirova-Ivanovska

**Affiliations:** 1 Department of Head and Neck Cancer, University Clinic of Radiotherapy and Oncology, Skopje, Macedonia; 2 Institute of Epidemiology, Statistics and Informatics, Faculty of Medicine, Skopje, Macedonia

**Keywords:** Concurrent radiochemotherapy, Oropharyngeal carcinoma, Prognostic factor

## Abstract

**Background:**

Concurrent radiochemotherapy is a recommended treatment option for patients with locally advanced squamous cell head and neck carcinomas with recent data showing the most significant absolute overall and event-free survival benefit achieved in patients with oropharyngeal tumours. The aim of this study was to analyse the results of three-dimensional conformal radiotherapy given with concomitant weekly cisplatin in patients with advanced oropharyngeal carcinoma and to identify prognostic factors influencing outcomes of this patients category.

**Methods:**

Sixty-five patients with stage III or IV squamous cell carcinoma of the oropharynx who underwent concurrent radiochemotherapy between January 2005 and December 2010 were retrospectively analyzed. All patients received radiotherapy to 70 Gy/35 fractions/2 Gy per fraction/5 fractions per week. Concurrent chemotherapy consisted of weekly cisplatin (30 mg/m^2^) started at the first day of radiotherapy.

**Results:**

Median age was 57 years (range, 36 to 69 years) and 59 (90.8%) patients were male. Complete composite response was achieved in 47 patients (72.3%). Local and/or regional recurrence was the most frequent treatment failure present in 19 out of 25 patients (76.0%). At a median follow-up of 14 months (range, 5 to 72 months), 2-year local relapse-free, regional relapse-free, locoregional relapse-free, disease-free, and overall survival rates were 48.8%, 57.8%, 41.7%, 33.2% and 49.7%, respectively.

On multivariate analysis the only significant factor for inferior regional relapse-free survival was the advanced N stage (p = 0.048). Higher overall stage was independent prognostic factor for poorer local relapse-free survival, locoregional relapse-free survival and disease-free survival (p = 0.022, p = 0.003 and p = 0.003, respectively). Pre-treatment haemoglobin concentration was an independent prognostic factor for local relapse-free survival, regional relapse-free survival, locoregional relapse-free survival, disease-free survival, and overall survival (p = 0.002, p = 0.021, p = 0.001, p = 0.002 and p = 0.002, respectively).

**Conclusions:**

Poor treatments results of this study suggested that introduction of intensity-modulated radiotherapy, use of induction chemotherapy followed by concurrent radiochemotherapy, accelerated radiotherapy regimens, and molecular targeted therapies could positively influence treatment outcomes. The incorporation of reversal of anaemia should be also expected to provide further improvement in locoregional control and survival in patients with advanced squamous cell carcinoma of the oropharynx.

## Background

Squamous cell carcinoma of the oropharynx is the eleventh most common cancer worldwide [1] with an annual incidence of 0.8 per 100000 [2]. Besides the strong association of oropharyngeal carcinoma with tobacco and alcohol abuse observed in epidemiological and clinical studies [3], the specific association of this cancer with human papillomavirus (HPV) infection has been now well-known [4,5]. The tonsil is the most frequently represented subsite of the primary oropharyngeal carcinoma followed by the base of tongue [6,7]. These two subsites account for between 80–90% of cases [2]. Oropharyngeal carcinomas are usually diagnosed as locoregionally advanced disease [8,9]. Thus, most of the primary tumours presents at an advanced stage (T2 or greater) [10], and the incidence of nodal metastases ranges between 60-70% [11,12] which is probably related to the rich lymphatic supply of the dominant subsites of the oropharyngeal cancers.

Treatment decision making process for oropharyngeal carcinomas arising in this functionally important anatomic region must take into consideration not only the most optimal treatment strategy for local/regional tumour control achievement but also the associated morbidity to this critical site in the upper aerodigestive tract. The treatment for advanced but resectable oropharyngeal carcinoma has traditionally been radical surgery and postoperative radiotherapy often resulting in suboptimal rates of locoregional control (LRC) and significant long-term functional deficits, or radiotherapy alone for advanced unresectable lesions inducing long-term toxicities accompanied with initial affection of speech and swallowing as a consequence of the primary tumour growth. However, in the last two decades, several randomized studies and meta-analyses indicated that concurrent radiochemotherapy (CRCT) has been shown to provide an improvement in LRC and survival as well as a significant increase in the rate of organ preservation when compared with radiation alone in patients with advanced head and neck cancer including those presented with advanced oropharyngeal carcinoma [13-22]. Apart from the trials including multiple sites in the head and neck cancers, the French Head and Neck Oncology and Radiotherapy Group (GORTEC 94-01) phase III randomized trial analyzing separately squamous cell carcinomas arising from the oropharynx established CRCT as the standard treatment for locoregionally advanced oropharyngeal cancer over conventional radiotherapy alone [23]. CRCT has been shown superior as compared with radiotherapy alone with regard to LRC, disease-free survival (DFS) and overall survival (OS) [23]. The final results of this trial reported in 2004 also confirmed that CRCT improved OS and LRC rates in patients with advanced oropharyngeal carcinoma [24]. Consequently, CRCT has been adopted as a preferred treatment approach that enables an achievement of increased rate of disease control with anatomic and physiologic function preservation [25]. Recently, in order to reveal the magnitude of the benefit of addition of chemotherapy to radiotherapy in terms of OS in head and neck squamous cell carcinoma according to tumour site, a comprehensive analysis was performed using individual data of 16,192 patients included in the meta-analysis of chemotherapy in head and neck cancer (MACH-NC) [26]. The interaction test showed the most significant effect of chemotherapy timing (p < 0.0001) in the oropharynx cancers group which was the largest group analysed consisting of 5878 patients, suggesting a significantly better effect of platinum-based concurrent chemotherapy [26]. Although the superiority of platinum-based chemotherapy has been confirmed in the meta-analysis of the MACH-NC Collaborative Group [19] and in the meta-analysis of Browman et al. [21], regarding the question about the number of chemotherapeutic agents and schedules of drug delivery, the optimum regimen seems to remain unclear. The most common method used worldwide was delivery of cisplatin every 3 weeks [17,27]. However, based on the assumption that more frequent drug administration could provide greater radiosensitizing benefit and taking into account the less induced morbidity with smaller individual doses of drug without compromising treatment efficacy [28], weekly administration of single-platinum agent has been also studied [29,30] although this schedules utilizing smaller doses more frequently has been not compared directly with the cyclical approach to delivery of concurrent cisplatin [31].

Regarding the radiotherapy techniques employed, it should be mentioned that the introduction of definitive three-dimensional conformal radiotherapy (3DCRT) with or without chemotherapy as the standard of practice in the treatment of oropharyngeal cancer in clinics around the world with tight target definitions of the primary tumour, metastatic nodes in the neck and neck nodal levels, enabled improvement of tumour coverage while sparing the surrounding critical tissues [32]. Recently, intensity-modulated radiotherapy (IMRT) achieving higher total doses in tumours by delivering larger doses per fraction to the tumour only, has also been shown as an effective treatment technique for locally advanced oropharyngeal carcinoma [33] offering tumour control rates kept at least at the level of 3DCRT while limiting dose to nearby normal tissues, e.g., the parotid glands [34,35]. Additionally, the Memorial Sloan-Kettering Cancer Centre experience and the University of California-San Francisco experience has revealed encouraging local control with acceptable treatment toxicity achieved using IMRT chemoradiation for treatment of stage III and IV oropharyngeal carcinoma [36,37]. The assessment of results of radiochemotherapy utilizing IMRT for advanced stage oropharyngeal carcinoma in the prospective study conducted by Feng et al. [38] demonstrated the possibility of this treatment approach to reduce post-therapy functional impairment obtaining at the same time high rates of locoregional tumour control.

The objective of this retrospective study was to summarize the results of treatment following 3DCRT and concomitant chemotherapy in patients with advanced oropharyngeal squamous cell carcinoma as well as to examine and identify the influence of various patient and tumour-related prognostic factors on local control, regional control, and survival in this patients population. We also considered that this retrospective analysis will give us an opportunity to compare results obtained with CRCT with those that would be achieved with IMRT whose implementation in the treatment of advanced oropharyngeal carcinomas at our institution has been started recently.

## Methods

This study is based on a retrospective analysis of 65 consecutive patients with previously untreated, stage III or IV primary squamous cell carcinoma of the oropharynx without distant metastases, at the age of at least 18 years and not more than 70 years and performance status 0 to 1, that underwent CRCT between January 2005 and December 2010 at the University Clinic of Radiotherapy and Oncology in Skopje.

Detailed patients evaluation prior to treatment included complete medical history with attention paid to disease-related signs and symptoms, and tobacco or alcohol abuse, clinical examination and fiberoptic endoscopy with biopsy, fine-needle aspiration biopsy in cases with detectable neck adenopathy, computed tomography (CT) scanning and/or magnetic resonance imaging (MRI) of head and neck region, chest x-ray, liver ultrasound, complete blood count, basic blood chemistry, and liver and renal function tests. Patients were staged according to the 2002 classification of the American Joint Committee on Cancer Staging (AJCC) [39].

### Treatment

3DCRT was performed on a linear accelerator using photons with beam qualities of 6 MV and 15 MV and electrons with energies 9-16 MeV. In patients with clinically negative neck the gross tumour volume (GTV) was represented by the gross tumour volume of the primary tumour (GTVt70) only and defined as any visible tumour revealed on imaging studies and/or physical examination. In patients with clinically positive neck the GTV was an union of GTVt70 and GTVn70. The GTVn70 was defined as the gross nodal disease revealed on imaging studies and/or physical examination. Neck lymph nodes were considered metastatic when their smallest axis diameter was greater than 1.0 cm. The clinical target volume (CTVt50) encompassed the GTVt70 plus a margin of 1.0-2.0 cm for the potential microscopic extension of the disease according to anatomical barriers. The CTVn50 encompassed the metastatic lymph node(s) if present plus at least 0.5-1.0 cm margins. This volume also included node levels in the neck according to the nodal status (bilateral level II, III and IV in patients with clinically negative neck, and bilateral level Ib, II, III, IV, V and retropharyngeal lymph nodes in patients with nodal disease). Retrostyloid space was also included in cases with positive lymph node(s) in level II. Delineation of the neck lymph node levels was realized according to Danish Head and Neck Cancer Group (DAHANCA), European Organization for Research and Treatment of Cancer (EORTC), Groupe d'Oncologie Radiothérapie Tête et Cou (GORTEC), National Cancer Instituteof Canada (NCIC), Radiation Therapy Oncology Group (RTOG) consensus guidelines [40] and proposals for the delineation of the nodal clinical target volume in the node positive and the postoperative neck [41]. CTV50 was created by integration of CTVt50 and CTVn50. The planning target volumes were PTV50 and PTV70. The PTV50 provided a margin of 0.5 cm around CTV50. The PTV70 encompassed the GTV plus a 0.5 cm margin. Conventional fractionation was used with a daily dose of 2.0 Gy, 5 times per week.

Chemotherapy was administered with radiation in concomitant setting. The regimen used consisted of weekly cisplatin (30 mg/m^2^) started at the first day of radiotherapy. Cisplatin was given before irradiation and the time gap between cisplatin administration and radiotherapy was no longer than three hours. Hydration and antiemetics were delivered according to standards of care. Complete blood count and biochemical analysis of serum urea and creatinine were done every week.

### Response assessment and follow-up

Evaluation of tumour response was performed 3 months after the completion of CRCT by physical examination, fiberoptic endoscopy, and CT or MRI of the primary site and the neck. Endoscopy under anaesthesia and biopsy of any clinical, endoscopic or radiological abnormality found was performed to reveal and confirm the suspicious residual lesion. Response to treatment was documented by the World Health Organization (WHO) response grading system [42]. Complete response of the primary tumour was defined as complete disappearance of all detectable disease at the primary site to visual inspection and imaging studies. Complete response of the nodal disease was defined as complete disappearance of all nodal disease on clinical examination and imaging studies. Complete composite response was defined as complete disappearance of locoregional disease. Partial response was defined as tumour reduction by at least 50% of the sum of the product of perpendicular diameters of all measurable lesions on endoscopy and imaging studies without any appearance of new lesions.

Patients were followed up every month over the first year after treatment, every 2 months in the second year after treatment, every 3 to 6 months in the third through the fifth years after treatment, and every 12 months thereafter. Each follow-up examination included history, physical examination, and fiberoptic endoscopy, or indirect mirror exam. Diagnostic imaging of the head and neck region was performed in any patient with signs and symptoms suggesting recurrence development with biopsy performed in order to obtain histological proof of clinically suspicious recurrent disease.

### Statistical analysis

All patients were included in the survival analysis. Statistical end points of this study were local relapse-free survival (LRFS), regional relapse-free survival (RRFS), locoregional relapse-free survival (LRRFS), DFS, and OS. LRFS for patients with complete response of the primary tumour was measured from the day of treatment start to the date when reappearance of primary disease was first recorded, or to the date of the last follow-up. For patients with persistent primary disease LRFS was measured from the first day of treatment to the date of the first follow-up visit. RRFS for patients with clinically negative neck and for those who achieved complete response of the nodal disease following treatment was measured from the day of treatment start to the date when appearance of metastatic lymph nodes in the neck or recurrence of the neck disease was first recorded, or to the date of the last follow-up. For patients with persistent nodal disease RRFS was measured from the first day of treatment to the date of the first follow-up visit. LRRFS for patients who achieved complete composite response to CRCT was measured from the first day of treatment to the date of reappearance of disease either at the primary site and/or regional lymph nodes, or until the day of the last follow-up. For patients initially staged as N0 who manifested complete primary response to treatment, LRRFS was calculated from the date of treatment beginning until the date when appearance of metastatic lymph node(s) in the neck and/or reappearance of disease at the primary site were first reported, or to the last follow-up date. DFS was calculated from the date of commencement of treatment to the date when local, regional, locoregional or distant failure was first recorded or, in the case of local and/or regional persistent disease, to the date of first follow-up visit. OS was measured from the start date of treatment to the date of the last follow-up or to the date of death from any cause. LRFS, RRFS, LRRFS, PFS and OS were calculated using the method of Kaplan-Meier [43].

Gender, age at diagnosis (≤ 50 years vs. > 50 years ), Eastern Cooperative Oncology Group (ECOG) performance status (0 vs. 1), cigarette smoking (non-smokers vs. current smokers), alcohol consumption (non-drinkers vs. current drinkers), subsite at the primary site (tonsil vs. base of tongue vs. soft palate vs. posterior pharyngeal wall), T stage (T2-3 vs. T4), N stage (N0-1 vs. N2-N3), overall stage (III vs. IVA-B), histological differentiation (well vs. moderate vs. poor), and haemoglobin concentration before treatment (≤ 12.5 g/dL vs. > 12.5 g/dL) were also assessed as potential prognostic factors investigating their impact on LRFS, RRFS, LRRFS, DFS, and OS using the log-rank test and p index. Cox's regression model was used for multivariate analysis. Statistical significance was defined as p-value less than 0.05. Multivariate analysis included those prognostic factors that had displayed p-value < 0.05 in the univariate analysis.

## Results

### Patient and tumour characteristics

There were 59 (90.8%) males and 6 (9.2%) females studied. Median age was 57 years (range, 36 to 69 years). Mean age was 56.4 years ± 8.33 SD. Forty four patients (67.7%) were presented with ECOG performance status 0 and 21 patients (32.3%) had ECOG performance status 1. Regarding the cigarette smoking status, more than four fifths of patients (83.1%) were current smokers while only 11 patients (16.9%) were considered non-smokers (patients who never smoked or those who quitted smoking more than 3 years ago). Likewise, regarding the drinking status, almost two thirds of patients (25/65 [61.5%]) were current alcohol drinkers while 25 patients (38.5%) were considered non-drinkers (patients who never drunk and those who quitted alcohol consumption more than 3 years ago). The level of haemoglobin > 12.5 g/dL before treatment commencement was measured in 45 patients (69.2%). Haemoglobin concentration ≤ 12.5 g/dL was present in 20 patients (30.8%). The subsites of the primary tumour treated were: tonsil 36 (55.4%), base of tongue 21 (32.3%), soft palate 6 (9.2%), and posterior pharyngeal wall 2 (3.1%). The distribution according to AJCC of overall stages was as follows: stage III 23 (35.4%), stage IVA 36 (55.4%), and stage IVB 6 (9.2%). The distribution of T and N stages as well as the distribution of degrees of histological differentiation is provided in Table 1.

**Table 1:** Tumour characteristics (n = 65)

**Characteristics**	**No. of patients**	**%**
Subsite of the primary tumour		
Tonsil	36	55,4
Base of tongue	21	32,3
Soft palate	6	9,2
Posterior pharyngeal wall	2	3,1
T stage		
T2	4	6,2
T3	43	66,1
T4	18	27,7
N stage		
N0	20	30,8
N1	14	21,5
N2	25	38,5
N3	6	9,2
Overall stage		
III	23	35,4
IVA	36	55,4
IVB	6	9,2
Histological differentiation		
Well	22	33,8
Moderate	25	38,5
Poor	18	27,7

### Compliance of treatment

All patients received the prescribed total radiotherapy dose (70 Gy). In vast majority of patients (55 patients, 84.6%), the overall treatment time for radiotherapy completion was ≤ 7 weeks. Ten patients (15.4%) required greater than 7 weeks to complete treatment with interruptions due to a variety of causes including comorbidity. Sixty per cent of patients completed all seven cycles of concurrent chemotherapy, and the remaining 40% received six cycles of concurrent weekly cisplatin. The mean total dose of cisplatin given was 192 mg/m^2^ ± 14.8 SD.

### Response to treatment

Complete response at the primary site was seen in 48 patients (73.8%). Complete response of the metastatic lymph node(s) in the neck occurred in 31 patients (68.9%). Complete composite response was present in 47 patients (72.3%). Partial composite response was registered in 18 patients (27.7%). Isolated residual disease at the primary site was seen in two patients and at the nodal site in only one patient. There was no salvage neck dissection performed for residual neck disease. Detailed data about complete response following treatment in accordance with the subsite of the primary tumour are listed in Table 2. There was an almost equal complete response rate at the primary site revealed in patients with carcinoma of the tonsil and carcinoma of the base of tongue whereas complete response rate of the nodal disease was slightly higher in patients with base of tongue cancer.

**Table 2:** Response to treatment in accordance with the subsite of the primary tumor

**Subsite of the primary tumour**	**Complete response of the primary tumour**	**Complete response of the nodal disease**	**Complete composite response**
Tonsil	26/36 (72.2%)	16/25 (64.0%)	25/36 (69.4%)
Base of tongue	16/21 (76.2%)	12/15 (80.0%)	16/21 (76.2%)
Soft palate	4/6 (66.7%)	2/4 (50.0%)	4/6 (66.7%)
Posterior wall	2/2 (100.0%)	1/1 (100.0%)	2/2 (100.0%)

### Patterns of failure

**Figure 1:**
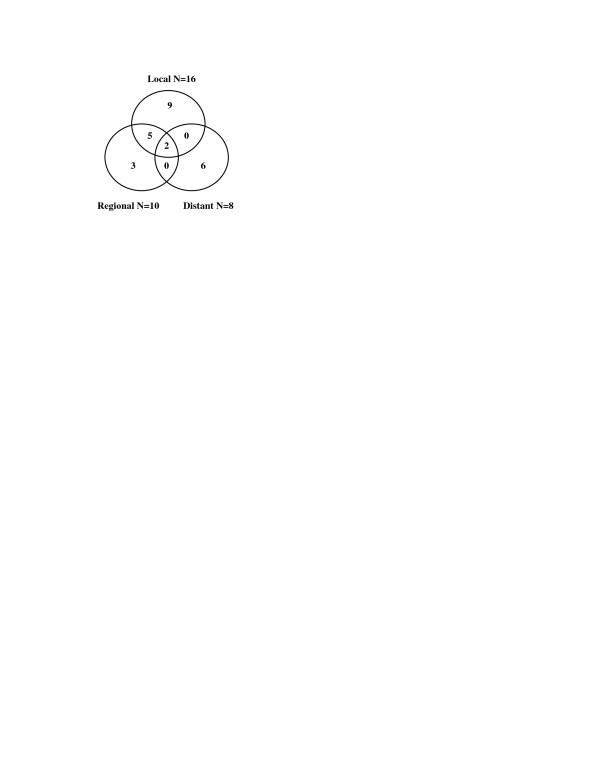
Venn diagram of patterns of failure.

The median follow-up was 14 months (range, 5 to 72 months) and 19 months (range, 10 to 72 months) for all and for living patients respectively. Treatment failure occurred in 25 of 47 patients (53.2%) with complete remission at three months post-treatment assessment. Local recurrence was noted in 9 patients, isolated regional recurrence was present in 3 patients, and 7 patients developed locoregional recurrence. Not one of the patients with local, regional, or locoregional recurrence was treated with salvage surgery. Distant metastases were detected in 8 patients with complete remission following CRCT. In two of these patients distant metastases development was preceded by occurrence of locoregional failure. Distant metastases were also manifested in 2 patients with persistent locoregional disease following treatment. The overall incidence of distant metastases was 15.4% (10/65). The lungs were the most frequent site of distant metastases (70%). The distribution of patterns of failure is illustrated in Figure 1. The median time to occurrence of local recurrence, regional recurrence, locoregional recurrence and distant metastases was 12 months (range 9-35), 20 months (range 10-22), 10 months (range 8-17) and 12 months (range 7-20 ), respectively.

### Survival

**Figure 2:**
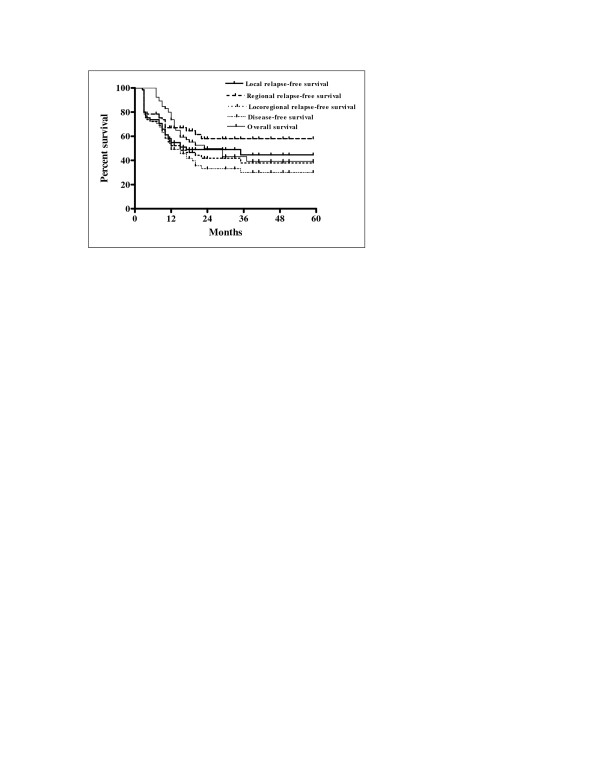
Local relapse-free survival, regional relapse-free survival, locoregional relapse-free survival, disease-free survival, and overall survival rates for all patients.

LRFS, RRFS, LRRFS, DFS, and OS rates at 2 years were: 48.8%, 57.8%, 41.7%, 33.2% and 49.7%, respectively (Figure 2). The median duration of LRFS and RRFS survival was 12 months (range 2.5-72) and 13 months (range 2.5-72), respectively. The median duration of LRRFS and DFS was 12 months (range 2.5-72), and the median duration of OS was 15 months (range 7-72).

There have been 32 deaths over the period of study: 18 were from the progression of local, regional, or locoregional persistent disease, 5 due to local recurrence, 2 due to regional recurrence, 4 due to recurrence at both primary and nodal site, 2 were due to distant metastases, and 1 due to both locoregional recurrence and distant metastases development. At the time of analysis 33 patients were alive. Twenty two of them were registered without disease at the last follow-up visit.

### Univariate analysis for prognostic factors

**Figure 3:**
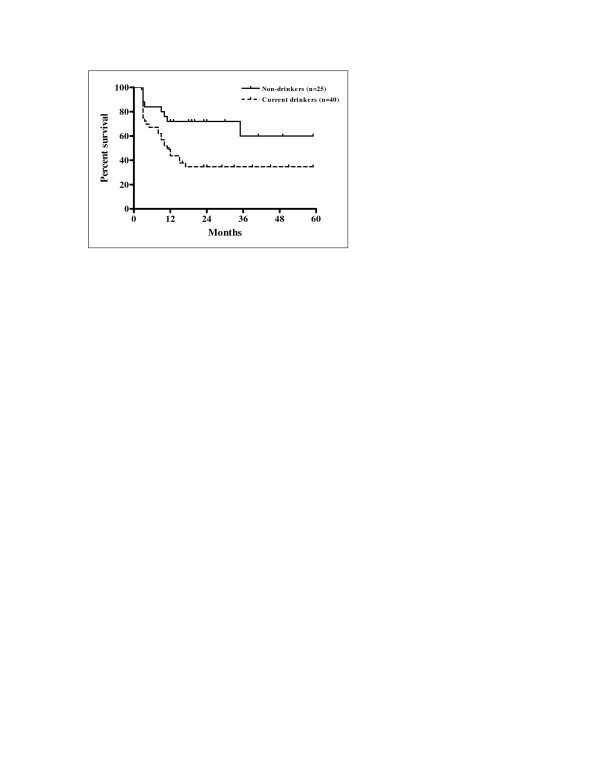
Local relapse-free survival according to alcohol consumption (Kaplan-Meier estimates) Log-rank test; Chi square = 5.773; p = 0.0163.

**Figure 4:**
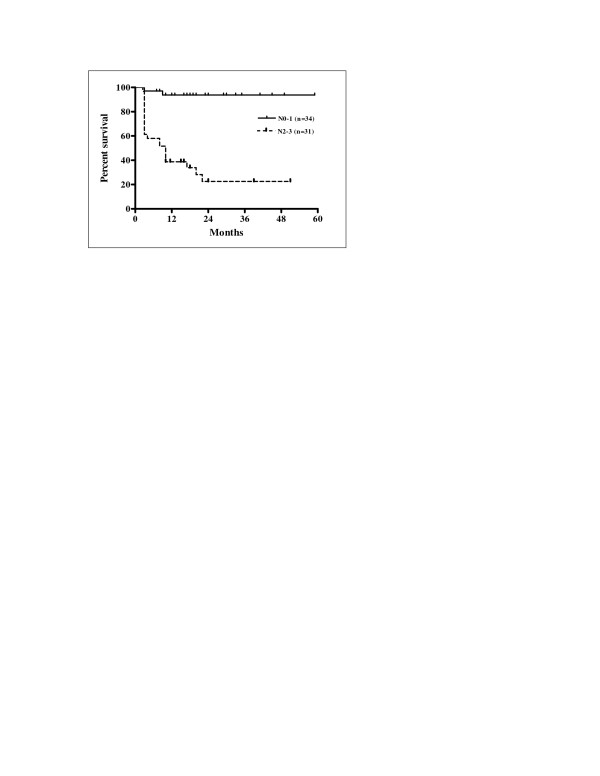
Regional relapse-free survival according to N stage (Kaplan-Meier estimates). Log-rank test; Chi square = 29.04; p < 0.0001.

**Figure 5:**
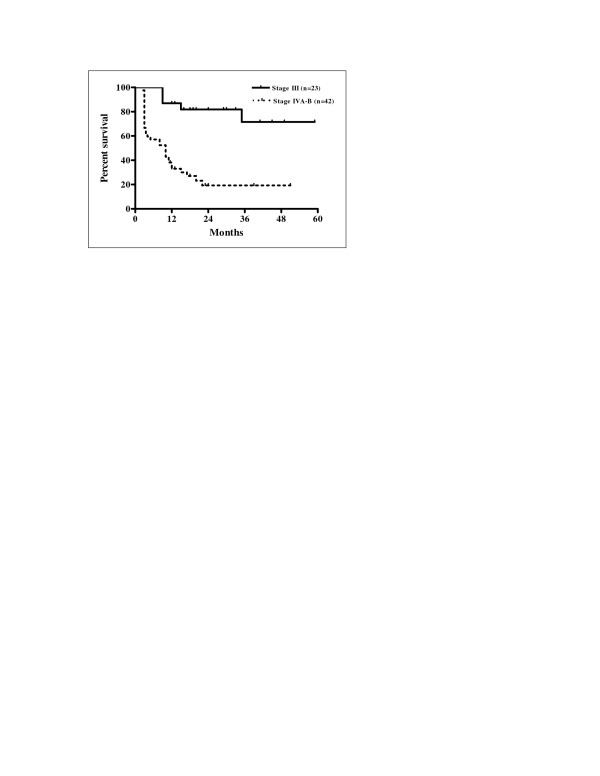
Locoregional relapse-free survival according to overall stage (Kaplan-Meier estimates) Log-rank test; Chi square = 18.88; p < 0.0001.

Univariate analysis of the eleven variables allowed the identification of factors significantly associated with prognosis. The results of univariate analysis for factors influencing LRFS, RRFS, and LRRFS are shown in Table 3. Statistically significant differences in LRFS, RRFS and LRRFS rates were associated with ECOG performance status, alcohol consumption, N stage, overall stage, and pre-treatment haemoglobin level. Cigarette smoking was revealed as a factor significantly associated with RRFS and LRRFS. There was a statistically significant difference in LRRFS associated with T stage. Patients with ECOG performance status 1 had significantly poorer LRFS, RRFS and LRRFS compared with patients with performance status ECOG 0 (p = 0.0004, p = 0.0169 and p = 0.0004, respectively). Current drinkers had significantly worse LRFS, RRFS and LRRFS compared with non-drinkers (p = 0.0163, p = 0.0222 and p = 0.0094, respectively). Kaplan-Meier curves of LRFS in relation with alcohol consumption are shown in Figure 3. Higher N stage (N2-3) had a significant negative influence on LRFS, RRFS and LRRFS in comparison with clinically negative neck and low N stage (N0-1) (p = 0.0006, p < 0.0001, and p < 0.0001, respectively). Kaplan-Meier curves of RRFS related to N stage are shown in Figure 4. Overall stage IVA-B had also significant negative impact on LRFS, RRFS and LRRFS compared with stage III disease (p = 0.0002, p < 0.0001, and p < 0.0001, respectively). Kaplan-Meier curves of LRRFS in relation with overall stage are depicted in Figure 5. Patients with pre-treatment haemoglobin level ≤ 12.5 g/dL had worse prognosis related to LRFS, RRFS and LRRFS compared with the group of patients with pre-treatment haemoglobin level > 12.5 g/dL (p < 0.0001, p = 0.0046 and p < 0.0001, respectively). The rates of RRFS and LRRFS were significantly lower in current smokers compared with non-smokers (p = 0.0365 and p = 0.0475, respectively). Primary lesions classified as T4 had significant negative influence on LRRFS in comparison with lesions classified as T2-3 (p = 0.0451).

**Table 3:** Univariate analysis correlating prognostic factors with disease control above clavicles

**Factor**		**LRFS**		**RRFS**		**LRRFS**	
	**n**	**2 years ** **%**	**p-value**	**2 years ** **%**	**p-value**	**2 years ** **%**	**p-value**
**Gender**							
Male	59	45.2	0,2713	52.3	0,0527	36.2	0,1562
Female	6	83.0		100.0		83.3	
**Age (years)**							
≤ 50	20	49.3	0,7046	42.3	0,3524	30.7	0,6735
> 50	45	48.2		63.2		44.8	
**Performance status (ECOG)**							
0	44	62.8	**0,0004**	68.3	**0,0169**	54.7	**0,0004**
1	21	19.2		20.8		9.8	
**Cigarette smoking**							
Non-smokers	11	77.7	0,1651	89.8	**0,0365**	79.9	**0,0475**
Current smokers	54	43.2		49.8		33.8	
**Alcohol consumption**							
Non-drinkers	25	71.8	**0,0163**	75.3	**0,0222**	64.7	**0,0094**
Current drinkers	40	34.8		47.2		27.2	
**Subsite**							
Tonsil	36	42.8	0,3656	58.7	0,7991	37.8	0.4509
Base of tongue	21	66.7		54.7		45.9	
Soft palate	6	49.2		50.2		50.9	
Posterior pharyngeal wall	2	0		0		0	
**T stage**							
T2-3	47	54.0	0,1094	58.8	0,9960	49.1	**0,0451**
T4	18	35.4		48.1		21.6	
**N stage**							
N0-1	34	68.8	**0.0006**	93.8	**<0.0001**	66.6	**<0.0001**
N2-3	31	28.3		22.2		16.4	
**Overall stage**							
III	23	81.7	**0.0002**	95.3	**<0.0001**	81.6	**<0.0001**
IVA-B	42	30.8		33.2		19.1	
**Histological differentiation**							
Well	22	57.2	0,1931	59.2	0,1385	44.1	0,2296
Moderate	25	54.3		76.3		54.7	
Poor	18	31.8		36.3		23.4	
**Haemoglobin level**							
≤ 12.5 g/dL	20	21.3	**<0.0001**	44.2	**0,0046**	19.0	**<0.0001**
> 12.5 g/dL	45	60.1		65.8		51.5	

n, number of patients; LRFS, local relapse-free survival; RRFS, regional relapse-free survival; LRRFS, locoregional relapse-free survival; ECOG, Eastern Cooperative Oncology Group.

**Figure 6:**
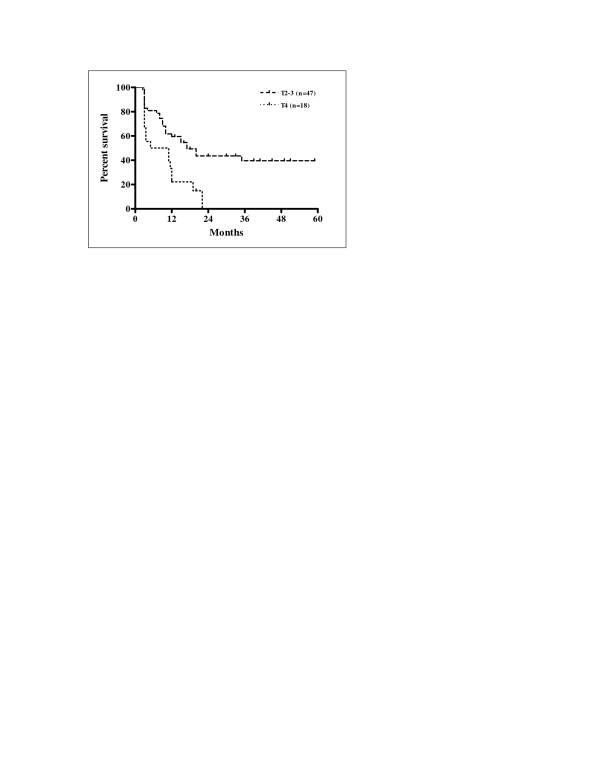
Disease-free survival according to T stage (Kaplan-Meier estimates) Log-rank test; Chi square = 8.610; p = 0.0033.

**Figure 7:**
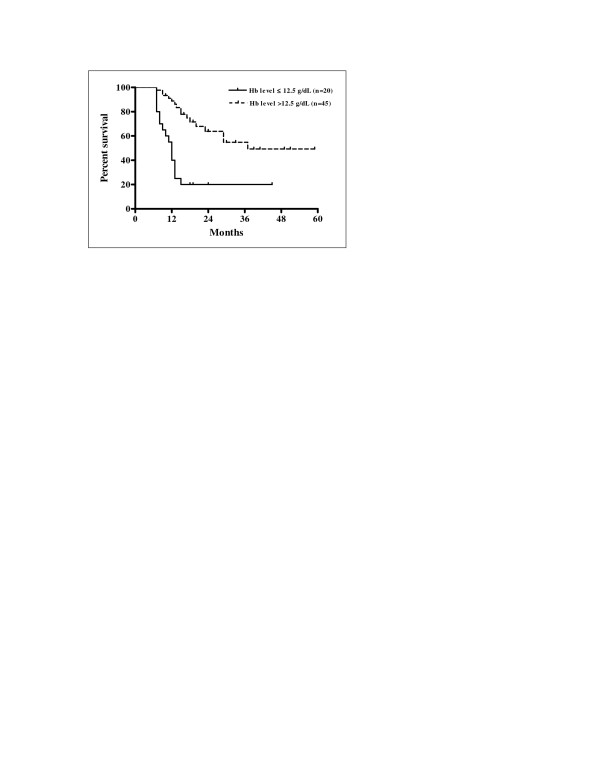
Overall survival according to pre-treatment haemoglobin concentration (Kaplan-Meier estimates) Log-rank test; Chi square = 19.66; p < 0.0001.

The impacts of the variables on DFS and OS are summarized in Table 4. The results of the univariate analysis revealed ECOG performance status, alcohol consumption, T stage, N stage, overall stage and pre-treatment haemoglobin level as significant prognostic factors for both DFS and OS whereas the smoking status was identified as a significant prognostic factor for DFS only. There were statistically significant lower rates of DFS and OS in patients with ECOG performance status 1 (p < 0.0001 for both), in those patients who were current drinkers (p = 0.0080 and p = 0.0333, respectively), in the group of patients with T4 primary lesion (p = 0.0033 and p = 0.0455, respectively), in patients with advanced nodal disease (N2-3) (p = 0.0004 for both), in those patients with overall stage IVA-B (p < 0.0001 and p = 0.0006, respectively), and in patients with pre-treatment haemoglobin level ≤ 12.5 g/do (p < 0.0001 for both). Statistically significant lower DFS was present in patients who were classified as current smokers (p = 0.323). Kaplan-Meier curves of DFS related to the T stage are shown in Figure 6. Kaplan-Meier curves of OS in relation with the pre-treatment haemoglobin concentration are shown in Figure 7.

**Table 4:** Univariate analysis correlating prognostic factors with disease-free and overall survival

**Factor**		**DFS**		**OS**	
	**n**	**2 years** **%**	**p-value**	**2 years** **%**	**p-value**
**Gender**					
Male	59	25.9	0,0711	45.4	0,1789
Female	6	83.4		83.3	
**Age (years)**					
≤ 50	20	27.8	0,8528	52.5	0,9873
> 50	45	34.7		49.8	
**Performance status (ECOG)**					
0	44	46.5	**<0.0001**	65.9	**<0.0001**
1	21	0		11.6	
**Cigarette smoking**					
Non-smokers	11	72.2	**0,0323**	72.8	0,1421
Current smokers	54	23.8		43.4	
**Alcohol consumption**					
Non-drinkers	25	56.5	**0,0080**	65.9	**0,0333**
Current drinkers	40	19.1		39.7	
**Subsite**					
Tonsil	36	29.3	0,4396	53.4	0,3679
Base of tongue	21	41.8		47.2	
Soft palate	6	0		50.3	
Posterior pharyngeal wall	2	0		0	
**T stage**					
T2-3	47	43.4	**0,0033**	57.2	**0,0455**
T4	18	0		39.7	
**N stage**					
N0-1	34	50.9	**0.0004**	73.4	**0.0004**
N2-3	31	14.1		26.6	
**Overall stage**					
III	23	70.3	**<0.0001**	80.3	**0.0006**
IVA-B	42	12.2		30.8	
**Histological differentiation**					
Well	22	40.9	0,1056	51.6	0,1287
Moderate	25	41.6		63.4	
Poor	18	10.3		32.2	
**Haemoglobin level**					
≤ 12.5 g/dL	20	14.7	**<0.0001**	19.7	**<0.0001**
> 12.5 g/dL	45	41.6		63.8	

n, number of patients; DFS, disease-free survival; OS, overall survival; ECOG, Eastern Cooperative Oncology Group.

### Multivariate analysis for prognostic factors

The results of multivariate analysis performed using the prognostic factors confirmed as significant in the univariate analysis are depicted in Table 5 and Table 6. The advanced N stage was revealed as an independent prognostic factor for inferior RRFS (N2-3 vs. N0-1, p = 0.048). The advanced overall stage was found to be an independent factor negatively influencing LRFS (IVA-B vs. III, p = 0.022), LRRFS (IVA-B vs. III, p = 0.003), and DFS (IVA-B vs. III, p = 0.003). Haemoglobin level was also independently prognostic for lower rates of LRFS (≤ 12.5 g/dL vs. > 12.5 g/dL, p = 0.002), RRFS (≤ 12.5 g/dL vs. > 12.5 g/dL, p = 0.021), LRRFS (≤ 12.5 g/dL vs. > 12.5 g/dL, p = 0.001), DFS (≤ 12.5 g/dL vs. > 12.5 g/dL, p = 0.002), and OS (≤ 12.5 g/dL vs. > 12.5 g/dL, p = 0.002).

**Table 5:** Multivariate analysis for local relapse-free survival, regional relapse-free survival and locoregional relapse-free survival

**Factors**	**Hazard ratio (95% CI)**		
	**LRFS**	**RRFS**	**LRRFS**
Performance status (ECOG) (1 vs. 0)	1.11 (0.48-2.55) p = 0.806	0.67 (0.25-1.82) p = 0.431	1.12 (0.50-2.54) p = 0.779
Cigarette smoking (current smokers vs. non-smokers)	/	3.98 (0.42-37.89) p = 0.229	0.61 (0.15-2.45) p = 0.485
Alcohol consumption (current drinkers vs. non-drinkers)	1.35 (0.57-3.20) p = 0.503	0.81 (0.25-2.63) p = 0.723	1.71 (0.61-4.84) p = 0.308
T stage (T4 vs. T2-3)	/	/	0.58 (0.22-1.52) p = 0.265
N stage (N2-3 vs. N0-1)	1.13 (0.44-2.89) p = 0.803	7.68 (1.02-58.05) **p = 0.048**	0.73 (0.23-2.25) p = 0.577
Overall stage (IVA-B vs. III)	4.49 (1.24-16.27) **p = 0.022**	3.57 (0.21-60.76) p = 0.379	9.91 (2.16-45.42) **p = 0.003**
Haemoglobin level ≤ 12.5 g/dL vs. > 12.5 g/dL	0.26 (0.11-0.62) **p = 0.002**	0.27 (0.09-0.82) **p = 0.021**	0.24 (0.10-0.57) **p = 0.001**

95% CI, 95% confidence interval; LRFS, local relapse-free survival; RRFS, regional relapse-free survival; LRRFS, locoregional relapse-free survival; ECOG, Eastern Cooperative Oncology Group.

**Table 6:** Multivariate analysis for disease-free survival and overall survival

**Factors**	**Hazard ratio (95% CI)**	
	**DFS**	**OS**
Performance status (ECOG) (1 vs. 0)	1.20 (0.56-2.60) p = 0.634	1.51 (0.59-3.86) p = 0.395
Cigarette smoking (current smokers vs. non-smokers)	0.93 (0.25-3.41) p = 0.909	/
Alcohol consumption (current drinkers vs. non-drinkers)	1.47 (0.59-3.63) p = 0.399	1.17 (0.49-2.77) p = 0.722
T stage (T4 vs. T2-3)	0.70 (0.28-1.72) p = 0.431	0.76 (0.29-2.02) p = 0.582
N stage (N2-3 vs. N0-1)	0.62 (0.22-1.74) p = 0.362	1.10 (0.30-4.11) p = 0.887
Overall stage (IVA-B vs. III)	8.40 (2.11-33.47) **p = 0.003**	3.82 (0.73-19.97) p = 0.112
Haemoglobin level ≤ 12.5 g/dL vs. > 12.5 g/dL	0.28 (0.13-0.61) **p = 0.002**	0.24 (0.10-0.60) **p = 0.002**

95% CI, 95% confidence interval; DFS, disease-free survival; OS, overall survival; ECOG, Eastern Cooperative Oncology Group.

## Discussion

Disease control and organ function preservation represent important goals of treatment in advanced oropharyngeal carcinomas. The increased use of combined treatment approach by addition of chemotherapy to radiotherapy either as induction chemotherapy [44,45] or concurrent chemotherapy [23,24] has been shown to result in improvement in LRC and OS in patients with stage III-IV oropharyngeal cancer. Radiotherapy given concurrently with chemotherapy is considered best established definitive treatment approach for anatomic and functional organ preservation in locally-regionally advanced lesions arising from oropharynx and other sites in the head and neck region [19-21,23,46].

Apart from the GORTEC 94-01 study [23,24] that documented the statistically significant survival benefit for CRCT and strongly supported its employment in the management of carcinoma of the oropharynx, few other randomized studies were conducted to evaluate the role of CRCT in the treatment of advanced oropharyngeal carcinoma [25,47,48]. In the randomized phase III trial (ORO 93-01) comparing conventionally fractionated radiotherapy vs. accelerated hyperfractionated radiotherapy vs. radiotherapy with conventional fractionation plus concomitant chemotherapy using carboplatin and 5-fluorouracil in patients with advanced oropharyngeal cancer, significantly superior 2-year DFS was found in the group treated with CRCT [47]. Although the long-term results of ORO 93-01 trial did not reveal any statistical significance in terms of LRC, relapse-free survival, and OS among groups treated with conventional fractionation, altered fractionation and CRCT, the authors concluded that considering the almost double increase in the 5-year LRC, relapse-free survival, and OS rates achieved with the use of CRCT, this combined treatment approach should be recommended for patients with advanced squamous cell carcinomas of the oropharynx [48]. The results of prospective phase II randomized single-centre study conducted by Sharma et al. [25] comparing radical radiotherapy with CRCT with seven doses of weekly cisplatin in patients with advanced carcinoma of the oropharynx and nasopharynx confirmed the superiority of CRCT over radiotherapy alone resulting in higher OS rates.

In the present study of CRCT using 3DCRT and concurrent weekly cisplatin the observed 2-year rates of LRFS, RRFS, LRRFS, DFS, and OS were 48.8%, 57.8%, 41.7%, 33.2% and 49.7%, respectively.

Calais et al. [23] reporting the results of GORTEC 94-01 trial in which conventionally fractionated radiotherapy was given with concurrent chemotherapy consisting of three cycles of carboplatin and 5-fluorouracil in patients with advanced-stage oropharynx carcinoma, revealed that 3-year DFS rate was 42%, and a rate of 3-year OS was 50%, while the observed rate of LRC was 66%. These figures are superior to those obtained at 2 years in our study. The 5-year analysis of the data of GORTEC 94-01 study reported by Denis et al. [24] confirming the significant improvement in LRC and survival among patients with stage III and IV squamous cell oropharyngeal carcinoma treated with CRCT compared with those treated with radiotherapy alone, showed 5-year LCR rate of 47.6%, specific disease-free survival rate of 26.6%, and OS rate of 22.4% in the combined therapy group.

In the prospective randomized trial conducted by Gupta et al. [30] comparing induction chemotherapy followed by CRCT with CRCT, 57 patients with locally advanced oropharyngeal carcinoma were treated with a total dose of irradiation between 65 and 70 Gy and concurrent low dose cisplatin (35 mg/m^2^ weekly). Despite the fact that this regimen of CRCT is comparable with that used in the present study, differences exist in the rates of LRC and survival in favour of the study of Gupta with reported 2-year rates of LRC, DFS, and OS of 83%, 59.5%, and 70%, respectively.

In the retrospective study of Kokubo et al. [49], the achieved 3-year cause specific survival rate was 83% for 14 patients treated with CRCT using weekly cisplatin at least 3 times. CRCT utilizing 3DCRT and concomitant S-1 as an oral fluoropyrimidine in 38 patients with oropharyngeal carcinoma resulted in 3-year LRC, distant metastases-free survival, DFS, and OS rates of 75%, 80%, 65%, and 80%, respectively [50]. These reported higher rates of disease control in comparison with the results of our study could be partially attributable to the inclusion of patients with stage I and II oropharyngeal cancer.

In the retrospective study of Lee et al. [51] comparing efficacy of IMRT with conventional radiotherapy using delayed accelerated concomitant boost radiotherapy in the setting of concurrent platinum-based chemotherapy for locally advanced oropharyngeal carcinoma, the implementation of IMRT given with concurrent administration of cisplatin every 3 to 4 weeks in 41 patients with stage III/IV disease led to 3-year local-progression-free, regional-progression-free, locoregional progression-free, distant-metastases-free, disease-free, and OS rates of 95%, 94%, 92%, 86%, 82%, and 91%, respectively. These results regarding both LRC and survival are remarkably superior to the results obtained in our study.

Reviewing treatment outcomes for stage III and IV oropharyngeal carcinoma treated with IMRT and concurrent platinum-based chemotherapy, de Arruda et al. [36] and Huang et al. [37] also reported much better results compared with the results of our study with respect to local, regional, LRC and survival. High rates of 3-year disease-free and locoregional recurrence-free survivals (88% and 96%, respectively) have been also achieved in the prospective study of Feng et al. [38] assessing results of radiochemotherapy utilizing IMRT and weekly chemotherapy with carboplatin and paclitaxel in 73 patients with stages III to IV oropharyngeal carcinoma.

In the present study we found dose delivery of both radiotherapy and chemotherapy quite satisfactory with full planned dose of 70 Gy administered in all treated patients and seven cycles of concurrent chemotherapy completed in 60% of patients. However, the use of single agent taxane-based CRCT for patients with advanced head and neck cancer resulting in good response rates and survival [52,53] has been shown as well tolerated regimen [54]. Implemented in the treatment of stage III-IV oropharyngeal carcinoma, concurrent taxane-based chemotherapy has provided superior compliance as compared with that observed in the present study. Thus, in the retrospective, single institution study of Fukada et al. [55] exploring taxane based CRCT for the treatment of locally advanced oropharyngeal or hypopharyngeal carcinoma, the 6-cycle program of weekly low-dose docetaxel-based concurrent chemotherapy was realized in 80% of patients while in the phase II trial (ECOG E2399 study), 90% of patients received at least five cycles of weekly paclitaxel even though the drug was administered in a concurrent setting following induction chemotherapy [45]. Nevertheless, in a recently published nonrandomized small sample size study comparing low dose weekly paclitaxel versus low dose weekly cisplatin given concurrently with conventionally fractionated radiotherapy in locally advanced head and neck, both regimens were found equally well tolerated [56].

Despite the noted changes in patterns of failure in patients with head and cancer by some authors [57,58], the results of our study with the observed local and/or regional recurrence in 19 of 25 patients who manifested treatment failure (76%) correspond with historical data showing locoregional failure as the major problem in disease control [23,24,29,58]. The reported data on distant failure in studies on CRCT for advanced oropharyngeal cancer range between 32% to 53% [23,24,29,58]. In our study, distant metastases were recognized in 8 out of 25 patients (32%) who manifested treatment failure at some period after the achieved complete remission.

Considering the impact of clinical factors on treatment outcomes, the prognostic analysis in our study revealed overall stage as an independent factor strongly correlated with LRC and survival. Stage IVA-B was found to negatively influence LRFS, LRRFS, and DFS. Multivariate analysis in the study of Denis et al. [24] also showed stage IV disease as an important factor for short survival. The results of multivariate analysis in the study of Ho et al. [59] have also presented overall clinical stage as one of the strongest factors for survival.

In the multivariate analysis of our study, higher nodal stage (N2-N3 vs. N0-N1) was shown as independent prognostic factor only for poorer RRFS. In the study of Fukada et al. [55], stage N2c-N3 disease was recognized as influencing only OS. Agarwal et al. [60] found advanced nodal disease independently prognostic for inferior LRC and DFS. N stage was found to be an independent significant factor for local, regional, LRC, DFS and OS in the study of Johansen et al. [61]. Similarly, Perez et al. [62] also showed that N stage was one of the most significant independent factors affecting local and/or regional tumour control and DFS.

The results of multivariate analysis in our study showed that low pre-treatment haemoglobin concentration (≤ 12.5 g/dL) was independent prognostic factor negatively influencing LRFS, RRFS, LRRFS, DFS, and OS. In the study of Johansen et al. [61], haemoglobin level was found to be significant only for local tumour control. Multivariate analysis in the study of Denis et al. [24] revealed low haemoglobin level as the most negative factor for LRC, DFS, and OS. These results correspond with the results of multivariate analysis in the study of Fukada et al. [55] identifying anaemia as a significant negative independent factor for LRC and survival. The review of Kumar [63] has also revealed a strong evidence that low pre-treatment haemoglobin concentration as powerful, statistically significant prognostic factor had negative impact on local control and survival in head and neck cancer patients treated with definitive radiotherapy. The impact of anaemia on locoregional tumour control and survival in patients with squamous cell carcinoma of the larynx and pharynx was confirmed in the large DAHANCA study in which the use of the radiosensitizer nimorazole in association with radiotherapy was shown to significantly improve LRC and disease-specific survival [64]. According to Becker et al. [65], low haemoglobin concentration as a factor with a negative impact on treatment outcomes was associated with reduced tumour oxygenation resulting in radioresistance in head and neck cancer. The analysis of the relationship between pre-treatment measurements of tumour oxygen tension (pO_2_) and LRC and survival provided evidence that tumour hypoxia was associated with a poor prognosis in patients with advanced head and neck squamous cell carcinoma treated with radiotherapy [66,67]. The investigation of Nordsmark and Overgaard [68] confirmed pre-treatment haemoglobin level and tumour hypoxia prognostic for locoregional tumour control emphasizing that low level of tumour oxygenation was shown the strongest independent prognostic indicator for locoregional tumour control after definitive radiotherapy in advanced head and neck cancers.

Although the recent re-analysis of a large randomized trial conducted by DAHANCA [69] suggested that HPV positive oropharyngeal cancers most probably would not benefit from any modification of tumour oxygenation, it should be pointed out that in conditions without available data regarding HPV status, the reversal of anaemia should be strongly considered in all patients with advanced oropharyngeal carcinoma in order to eliminate the negative reflection of low pre-treatment haemoglobin level on the patient’s general condition and tumour progression [63,70].

Considering the established importance of HPV, expressed by p16, as a strong independent prognostic factor for survival among patients with oropharyngeal cancer [71], and taking into account published data indicating HPV-positivity as consistent determinant of superior survival irrespectively of the treatment approach used [69,70,72,73] it could be admitted that specific testing for HPV in oropharyngeal cancers is highly recommendable since the combination of HPV status and overall disease stage could be useful in further classification of patients providing in that way treatment decisions for individual patient moreover that the optimal treatment regimen for HPV/p16 positive oropharyngeal cancer has not been yet clarified.

## Conclusions

Our study, presenting single centre treatment experience, demonstrated inferior results in patient outcomes following single-agent weekly cisplatin radiochemotherapy regimen compared with the results of studies evaluating CRCT using the same or different scheduling of concomitant cisplatin regardless of the radiation technique used. Results of our study, especially when taking into consideration the highest proportion of local/regional relapse in the whole number of recurrences, do suggest that further progress in the management of this disease in our institution could be achieved by introduction of advanced radiotherapy techniques (IMRT) as an attempt to reduce incidence of locoregional failures and to influence improvement of DFS and OS rates. However, the high percentage of patients with distant metastatic development with or without synchronous manifestation of locoregional recurrence recognized in studies of CRCT should not be neglected and should point out the importance of further investigation of combined treatment approach represented with induction chemotherapy followed by CRCT. Despite all uncertainties of radiotherapy, the use of accelerated radiotherapy regimens which can lead to redefinition of treatment protocols and reorganization of patient and staff flow in the department, can lead to improvement of treatment outcome. Also, introduction of other cytotoxic agents (taxanes) in concurrent setting with conventional fractionated radiotherapy and the inclusion of molecular targeted therapies are expected to provide further improvement in treatment outcomes in patients with advanced squamous cell carcinoma of the oropharynx.

## Abbreviations

HPV, Human papillomavirus; LRC, Locoregional control; CRCT, Concurrent radiochemotherapy; DFS, Disease-free survival; OS, Overall survival; MACH-NC, Meta-Analysis of Chemotherapy in Head and Neck Cancer; 3DCRT, Three-dimensional conformal radiotherapy; IMRT, Intensity-modulated radiotherapy; CT, Computed tomography; MRI, Magnetic resonance imaging; AJCC, American Joint Committee on Cancer; GTV, Gross Tumour Volume; CTV, Clinical Target Volume; DAHANCA, Danish Head and Neck Cancer Group; EORTC, European Organization for Research and Treatment of Cancer; GORTEC, Groupe d'Oncologie Radiothérapie Tête et Cou; NCIC, National Cancer Institute of Canada; RTOG, Radiation Therapy Oncology Group; PTVs, Planning Target Volumes; WHO, World Health Organization; LRFS, Local relapse-free survival; RRFS, Regional relapse-free survival; LRRFS, Locoregional relapse-free survival; ECOG, Eastern Cooperative Oncology Group.

## Competing interests

The authors declare that they have no competing interests.

## Authors’ contributions

VK and IS have made substantial contributions to design of the study and analysed the data. VK collected the data and created the data base. VK performed much of the work and drafted the manuscript. VK and BI-Z performed the statistical analysis. VK and IS interpreted the data. All authors read and approved the final manuscript.

## References

[B1] Global data on incidence of oral cancer, Oral Health Programme World Health Organization[http://www.who.int/oral_health/publications/oral_cancer_brochure.pdf]

[B2] Evans PHR Patel SG Henk JM Evans PHR, Montgomery PQ, Gullane PJ Tumours of the oropharynx In Principles and Practice in Head and Neck Oncology. 2nd edition 2006 Taylor & Francis e-Library, London 376 437

[B3] Brugere J Guenel P Leclerc A Rodriguez J Differential effects of tobacco and alcohol in cancer of the larynx, pharynx, and mouth Cancer 1986 57 391 395 10.1002/1097-0142(19860115)57:2<391::AID-CNCR2820570235>3.0.CO;2-Q 3942973

[B4] Ringstrom E Peters E Hasegawa M Posner M Liu M Kelsey KT Human papillomavirus type 16 and squamous cell carcinoma of the head and neck Clin Cancer Res 2002 8 3187 3192 12374687

[B5] Gillison ML D’Souza G Westra W Sugar E Xiao W Begum S Viscidi R Distinct risk factor profiles for human papillomavirus type 16-positive and human papillomavirus 16-negative head and neck cancers J Natl Cancer Inst 2008 100 407 420 10.1093/jnci/djn025 18334711

[B6] Forastiere A Koch W Trotti A Sidransky D Head and neck cancer N Engl J Med 2001 345 1890 1900 10.1056/NEJMra001375 11756581

[B7] Ruff T Lenis A Skinner OD Carcinoma of the oral cavity and oropharynx Surg Clin North Am 1986 66 659 671 3738692 10.1016/s0039-6109(16)43979-4

[B8] Machtay M Perch S Markiewicz D Thaler E Chalian A Goldberg A Kligerman M Weinstein G Combined surgery and postoperataive radiotherapy for carcinoma of the base of tongue Head Neck 1997 19 494 499 10.1002/(SICI)1097-0347(199709)19:6<494::AID-HED6>3.0.CO;2-U 9278757

[B9] Carvalho AL Magrin J Kowalski LP Sites of recurrence in oral and oropharyngeal cancers according to the treatment approach Oral Dis 2003 9 112 118 10.1034/j.1601-0825.2003.01750.x 12945592

[B10] Guggenheimer J Verbin RS Johnson JT Horkowitz CA Myers EN Factors delaying the diagnosis of oral and oropharyngeal carcinomas Cancer 1989 64 932 935 10.1002/1097-0142(19890815)64:4<932::AID-CNCR2820640428>3.0.CO;2-Y 2743284

[B11] Henk JM Results of radiotherapy for carcinoma of the oropharynx Clin Otolaryngol & Allied Sci 1978 3 137 143 10.1111/j.1365-2273.1978.tb00676.x 668171

[B12] Lindberg R Distribution of cervical lymph node metastases from squamous cell carcinoma of the upper respiratory and digestive tracts Cancer 1972 29 1446 1450 10.1002/1097-0142(197206)29:6<1446::AID-CNCR2820290604>3.0.CO;2-C 5031238

[B13] Browman GP Cripps C Hodson DI Eapen L Sathya J Levine MN Placebo-controlled randomized trial of infusional fluorouracil during standard radiotherapy in locally advanced head and neck cancer J Clin Oncol 1994 12 2648 2653 7989940 10.1200/JCO.1994.12.12.2648

[B14] Haffty BG Son YH Papac R Sasaki CT Weissberg JB Fischer D Rockwell S Sartorelli AC Fischer JJ Chemotherapy as an adjunct to radiation in the treatment of squamous cell carcinoma of the head and neck: Results of the Yale Mitomycin Randomized Trials J Clin Oncol 1997 15 268 276 8996152 10.1200/JCO.1997.15.1.268

[B15] Wendt TG Grabenbauer GG Rodel CM Thiel HJ Aydin H Rohloff R Wustrow TP Iro H Popella C Schalhorn A Simultaneous radiochemotherapy versus radiotherapy alone in advanced head and neck cancer: a randomized multicenter study J Clin Oncol 1998 16 1318 1324 9552032 10.1200/JCO.1998.16.4.1318

[B16] Brizel DM Albers ME Fisher SR Scher RL Richtsmeier WJ Hars V George SL Huang AT Prosnitz LR Hyperfractionated irradiation with or without concurrent chemotherapy for locally advanced head and neck cancer N Engl J Med 1998 338 1798 1804 10.1056/NEJM199806183382503 9632446

[B17] Adelstein DJ Lavertu P Saxton JP Secic M Wood BG Wanamaker JR Eliachar I Strome M Larto MA Mature results of a phase III randomized trial comparing chemoradiotherapy with radiation therapy alone in patients with stage III and IV squamous cell carcinoma of the head and neck Cancer 2000 88 876 883 10.1002/(SICI)1097-0142(20000215)88:4<876::AID-CNCR19>3.0.CO;2-Y 10679658

[B18] Jeremic B Shibamoto Y Milicic B Nikolic N Dagovic A Aleksandrovic J Vaskovic Z Tadic L Hyperfractionated radiation therapy with or without concurrent low-dose daily cisplatin in locally advanced squamous cell carcinoma of the head and neck: a prospective randomized trial J Clin Oncol 2000 18 1458 1464 10735893 10.1200/JCO.2000.18.7.1458

[B19] Pignon JP Bourhis J Domenge C Designe L Chemotherapy added to locoregional treatment for head and neck squamous-cell carcinoma: three meta analyses of updated individual data. MACH-NC Collaborative Group. Meta-analysis of chemotherapy on head and neck cancer Lancet 2000 355 949 955 10768432

[B20] Pignon JP le Maitre A Maillard E Bourhis J Meta-analysis of chemotherapy in head and neck cancer (MACH-NC): an update on 93 randomized trials and 17,346 patients Radiother Oncol 2009 92 4 14 10.1016/j.radonc.2009.04.014 19446902

[B21] Browman GP Hodson DI Mackenzie RJ Bestic N Zuraw L Cancer Care Ontario Practice Guideline Initiative Head and Neck Cancer Disease Site Group Cancer Care Ontario Practice Guideline Initiative Head and Neck Cancer Disease Site Group Choosing a concomitant chemotherapy and radiotherapy regimen for squamous cell head and neck cancer: a systematic review of the published literature with subgroup analysis Head Neck 2001 23 579 589 10.1002/hed.1081 11400247

[B22] Budach W Hehr T Budach V Belka C Dietz K A Meta-Analysis of hyperfractionated and accelerated radiotherapy and combined chemotherapy and radiotherapy regimens in unresected locally advanced squamous cell carcinoma of the head and neck BMC Cancer 2006 6 28 39 10.1186/1471-2407-6-28 16448551 PMC1379652

[B23] Calais G Alfonsi M Bardet E Sire C Germain T Bergerot P Rhein B Tortochaux J Oudinot P Bertrand P Randomized trial of radiation therapy versus concomitant chemotherapy and radiation therapy for advanced-stage oropharynx carcinoma J Natl Cancer Inst 1999 91 2081 2086 10.1093/jnci/91.24.2081 10601378

[B24] Denis F Garaud P Bardet E Alfonsi M Sire C Germain T Bergerot P Rhein B Tortochaux J Calais G Final results of the 94-01 French Head and Neck Oncology and Radiotherapy group randomized trial comparing radiotherapy alone with concomitant radiochemotherapy in advanced-stage oropharynx carcinoma J Clin Oncol 2004 22 69 76 14657228 10.1200/JCO.2004.08.021

[B25] Sharma A Mohanti BK Thakar A Bahadur S Bhasker S Concomitant chemoradiation versus radical radiotherapy in advanced squamous cell carcinoma of oropharynx and nasopharynx using weekly cisplatin: a phase II randomized trial Ann Oncol 2010 21 2272 2277 10.1093/annonc/mdq219 20427350

[B26] Blanchard P Baujat B Holostenco V Bourredjem A Baey C Bourhis J Pignon J-P on behalf of the MACH-CH Collaborative group on behalf of the MACH-CH Collaborative group Meta-analysis of chemotherapy in head and neck cancer (MACH-HC: A comprehensive analysis by tumour site Radiother Oncol 2011 100 33 40 10.1016/j.radonc.2011.05.036 21684027

[B27] Adelstein DJ Li Y Adams GL Wagner H Kish JA Ensley JF Schuller DE Forastiere AA An intergroup phase III comparison of standard radiation and two schedules of concurrent chemoradiotherapy in patients with unresectable squamous cell head and neck cancer J Clin Oncol 2003 21 92 98 10.1200/JCO.2003.01.008 12506176

[B28] Kurihara N Kubota T Hoshiya Y Otani Y Ando N Kumai K Kitajima M Pharmacokinetics of cis-diamminedichloroplatinum (II) given as low-dose and high-dose infusions J Surg Oncol 1996 62 135 138 10.1002/(SICI)1096-9098(199606)62:2<135::AID-JSO10>3.0.CO;2-7 8649040

[B29] Gupta T Agarwal JP Ghosh-Laskar S Parikh PM D’Cruz AK Dinshaw KA Radical radiotherapy with concurrent weekly cisplatin in locoregionally advanced squamous cell carcinoma of the head and neck: a single-institution experience Head Neck Oncol 2009 1 17 10.1186/1758-3284-1-17 19527507 PMC2702367

[B30] Gupta D Shukla P Bisht SS Dhawan A Pant MC Bhatt ML Gupta S Gupta R Negi MPS A prospective comparision of sequential chemoradiation vs concurrent chemoradiation in locally advanced oropharyngeal carcinomas Cancer Biol Ther 2009 8 213 217 10.4161/cbt.8.3.7484 19276670

[B31] Brizel DM Esclamado R Concurrent chemoradiotherapy for locally advanced, nonmetastatic, squamous carcinoma of the head and neck: consensus, controversy, and conundrum J Clin Oncol 2006 24 2612 2617 10.1200/JCO.2005.05.2829 16763273

[B32] Purdy A Dose to normal tissues outside the radiation therapy patient's treated volume: a review of different radiation therapy techniques Health Phys 2008 5 666 676 18849701 10.1097/01.HP.0000326342.47348.06

[B33] Chao KS Ozyigit G Blanco AI Thorstad WL Deasy JO Haughey BH Spector GJ Sessions DG Intensity-modulated radiation therapy for oropharyngeal carcinoma: impact of tumor volume Int J Radiat Oncol Biol Phys 2004 59 43 50 10.1016/j.ijrobp.2003.08.004 15093897

[B34] Eisbruch A Ship JA Dawson LA Kim HM Bradford CR Terrell JE Chepeha DB Teknos TN Hogikyan ND Anzai Y Marsh LH Ten Haken RK Wolf GT Salivary gland sparing and improved target irradiation by conformal and intensity modulated irradiation of head and neck cancer World J Surg 2003 27 832 837 10.1007/s00268-003-7105-6 14509515

[B35] Studer G Huguenin PU Davis JB Kunz G Lutolf UM Glanzmann C IMRT using simultaneously integrated boost (SIB) in head and neck patients Radiat Oncol 2006 1 7 21 10.1186/1748-717X-1-7 16722599 PMC1459185

[B36] de Arruda FF Puri DR Zhung J Narayana A Wolden S Hunt M Stambuk H Pfister D Kraus D Shaha A Shah J Lee NY Intensity modulated radiation therapy for the treatment of oropharyngeal carcinoma: the Memorial Sloan-Kettering Cancer Center experience Int J Radiat Oncol Biol Phys 2005 64 363 373 15925451 10.1016/j.ijrobp.2005.03.006

[B37] Huang K Xia P Chuang C Weinberg V Glastonbury CM Eisele DW Lee NY Yom SS Phillips TL Quivey JM Intensity-modulated chemoradiation for treatment of stage III and IV oropharyngeal carcinoma: the University of California-San Francisco experience Cancer 2008 113 497 507 10.1002/cncr.23578 18521908

[B38] Feng FY Kim HM Lyden TH Haxer MJ Worden FP Feng M Moyer JS Prince ME Carey TE Wolf GT Bradford CR Chepeha DB Eisbruch A Intensity-modulated chemoradiotherapy aiming to reduce dysphagia in patients with oropharyngeal cancer: clinical and functional results J Clin Oncol 2010 28 2732 2738 10.1200/JCO.2009.24.6199 20421546 PMC2881852

[B39] Greene FL Page DL Fleming ID Fritz A Balch CM Haller DG Morrow M AJCC Cancer Staging Manual New York: Springer-Verlag 2002 6 33 46

[B40] Gregoire V Levendag P Ang KK Bernier J Braaksma M Budach V Chao C Coche E Cooper JS Cosnard G Eisbruch A El-Sayed S Emami B Grau C Hamoir M Lee N Maingon P Muller K Reychler H CT-based delineation of lymph node levels related CTVs in the node negative neck: DAHANCA, EORTC, GORTEC, NCIC, RTOG consensus guidelines Radiother Oncol 2003 69 227 236 10.1016/j.radonc.2003.09.011 14644481

[B41] Gregoire V Eisbruch A Hamoir M Levendag P Proposal for the delineation of the nodal CTV in the node-positive and post-operative neck Radiother Oncol 2006 79 15 20 10.1016/j.radonc.2006.03.009 16616387

[B42] Miller AB Hoogstraten B Staquet M Winkler A Reporting results of cancer treatment Cancer 1981 47 207 214 10.1002/1097-0142(19810101)47:1<207::AID-CNCR2820470134>3.0.CO;2-6 7459811

[B43] Kaplan EL Meier P Nonparametric estimation for incomplete observation J Am Stat Assoc 1958 53 457 481 10.1080/01621459.1958.10501452

[B44] Domenge C Hill C Lefebre JL De Raucourt D Rhein B Wibault P Marandas P Coche-Dequeant B Stromboni-Luboinski M Sancho-Garnier H Luboinski B Randomized trial of neoadjuvant chemotherapy in oropharyngeal carcinoma. French Groupe d’Etude des Tumeurs de la Tête et du Cou (GETTEC) Br J Cancer 2000 83 1594 1598 10.1054/bjoc.2000.1512 11189100 PMC2363468

[B45] Cmelak AJ Li S Goldwasser MA Murphy B Cannon M Pinto H Rosenthal DI Gillison M Forastiere AA Phase II trial of chemoradiation for organ preservation in resectable stage III or IV squamous cell carcinomas of the larynx or oropharynx: results of Eastern Cooperative Oncology Group Study E2399 J Clin Oncol 2007 25 3971 3977 10.1200/JCO.2007.10.8951 17761982

[B46] Forastiere AA Trotti A Radiotherapy and concurrent chemotherapy: a strategy that improves locoregional control and survival in oropharyngeal cancer J Natl Cancer Inst 1999 91 2065 2066 10.1093/jnci/91.24.2065 10601369

[B47] Olmi P Crispino S Fallai C Torri V Rossi F Bolner A Amichetti M Signor M Taino R Sguadrelli M Colombo A Ardizzoia A Ponticelli P Franchin G Minatel E Gobitti C Atzeni G Gava A Flann M Marsoni S Locoregionally advanced carcinoma of the oropharynx: conventional radiotherapy vs. accelerated hyperfractionated radiotherapy vs. concomitant radiotherapy and chemotherapy-a multicenter randomized trial Int J Radiat Oncol Biol Phys 2003 55 78 92 10.1016/S0360-3016(02)03792-6 12504039

[B48] Fallai C Bolner A Signor M Gava A Franchin G Ponticelli P Taino R Rossi F Ardizzoia A Oggionni M Crispino S Olmi P Long-term results of conventional radiotherapy versus accelerated hyperfractionated radiotherapy versus concomitant radiotherapy and chemotherapy in locoregionally advanced carcinoma of the oropharynx Tumori 2006 92 41 54 16683383 10.1177/030089160609200108

[B49] Kokubo M Nagata Y Nishimura Y Kimura H Shoji K Asato R Sasai K Hiraoka M Concurrent chemoradiotherapy for oropharyngeal carcinoma Am J Clin Oncol 2001 24 71 76 10.1097/00000421-200102000-00013 11232954

[B50] Ohnishi K Shioyama Y Nakamura T Ohga S Nonoshita T Yoshitake T Terashima K Komune S Honda H Concurrent chemoradiotherapy with S-1 as first-line treatment for patients with oropharyngeal cancer J Radiat Res 2011 52 47 53 10.1269/jrr.10081 21187666

[B51] Lee NY de Arruda FF Puri DR Wolden SL Narayana A Mechalakos J Venkatraman ES Kraus D Shaha A Shah JP Pfister DG Zelefsky MJ A comparison of intensity-modulated radiation therapy and concomitant boost radiotherapy in the setting of concurrent chemotherapy for locally advanced oropharyngeal carcinoma Int J Radiat Oncol Biol Phys 2006 66 966 974 10.1016/j.ijrobp.2006.06.040 17145527

[B52] Lovey J Koronczay K Remenar E Csuka O Nemeth G Radiotherapy and concurrent low-dose paclitaxel in locally advanced head and neck cancer Radiother Oncol 2003 68 171 174 10.1016/S0167-8140(03)00199-3 12972312

[B53] Suzuki M Nishimura Y Nakamatsu K Kanamori S Koike R Kawamoto M Mori K Phase I study of weekly docetaxel infusion and concurrent radiation therapy for head and neck cancer Jpn J Clin Oncol 2003 33 297 301 10.1093/jjco/hyg054 12913084

[B54] Pergolizzi S Adamo V Ferraro G Sergi C Santacaterina A Romeo A De Renzis C Zanghi M Rossello R Settineri N Induction chemotherapy to weekly paclitaxel concurrent with curative radiotherapy in stage IV (M0) unresectable head and neck squamous cell carcinoma: a dose escalation study J Chemother 2004 16 201 205 15216957 10.1179/joc.2004.16.2.201

[B55] Fukada J Shigematsu N Takeda A Ohashi T Tomita T Shiotani A Kuneida E Kawaguchi O Fujii M Kubo A Weekly low-dose docetaxel-based chemoradiotherapy for locally advanced oropharyngeal or hypopharyngeal carcinoma: a retrospective, single institution study Int J Radiat Oncol Biol Phys 2010 76 417 424 10.1016/j.ijrobp.2009.01.056 19409727

[B56] Jain RK Kirar P Gupta G Dubey S Gupta SK Goyal J A comparative study of low dose weekly paclitaxel versus cisplatin with concurrent radiation in the treatment of locally advanced head and neck cancer Indian J Cancer 2009 46 50 53 10.4103/0019-509X.48596 19282567

[B57] Vokes EE Kies MS Haraf DJ Stenson K List M Humerickhouse R Dolan ME Pelzer H Sulzen L Witt ME Hsieh YC Mittal BB Weichselbaum RR Concomitant chemoradiotherapy as primary treatment for locoregionally advanced head and neck cancer J Clin Oncol 2000 18 1652 1661 10764425 10.1200/JCO.2000.18.8.1652

[B58] Machtay M Rosenthal DI Hershock D Jones H Williamson S Greenberg MJ Weinstein GS Aviles VM Chalian AA Weber RS Organ preservation therapy using induction plus concurrent chemoradiation for advanced resectable oropharyngeal carcinoma: A University of Pennsylvania phase II trial J Clin Oncol 2002 20 3964 3971 10.1200/JCO.2002.11.026 12351593

[B59] Ho T Zahurak M Koch WM Prognostic significance of presentation-to-diagnosis interval in patients with oropharyngeal carcinoma Arch Otolaryngol Head Neck Surg 2004 130 45 51 10.1001/archotol.130.1.45 14732767

[B60] Agarwal JP Mallick I Bhutani R Ghosh-Laskar S Gupta T Budrukkar A Murthy V Sengar M Dinshaw KA Prognostic factors in oropharyngeal cancer-analysis of 627 cases receiving definitive radiotherapy Acta Oncol 2009 48 1026 1033 10.1080/02841860902845839 19363712

[B61] Johansen LV Grau C Overgaard J Squamous cell carcinoma of the oropharynx-an analysis of treatment results in 289 consecutive patients Acta Oncol 2000 39 985 994 10.1080/02841860050215981 11207007

[B62] Perez CA Patel MM Chao KS Simpson JR Sessions D Spector GJ Haughey B Lockett MA Carcinoma of the tonsillar fossa: prognostic factors and long-term therapy outcome Int J Radiat Oncol Biol Phys 1998 42 1077 1084 10.1016/S0360-3016(98)00291-0 9869232

[B63] Kumar P Impact of anemia in patients with head and neck cancer Oncologist 2000 5 Suppl 2 13 18 10.1634/theoncologist.5-suppl_2-13 10896324

[B64] Overgaard J Hansen HS Overgaard M Bastholt L Berthelsen A Specht L Lindelov B Jorgensen K A randomized double-blind phase III study of nimorazole as hypoxic radiosensitizer of primary radiotherapy in supraglottic larynx and pharynx carcinoma. Results form the Danish Head and Neck Cancer Study (DAHANCA). Protocol 5-85 Radiother Oncol 1998 46 135 146 10.1016/S0167-8140(97)00220-X 9510041

[B65] Becker A Stadler P Lavey RS Hansgen G Kuhnt T Lautenschlager C Feldmann HJ Molls M Dunst J Severe anemia is associated with poor tumor oxygenation in head and neck squamous cell carcinomas Int J Radiat Oncol Biol Phys 2000 46 459 466 10.1016/S0360-3016(99)00384-3 10661354

[B66] Nordsmark M Overgaard J A confirmatory prognostic study on oxygenation status and loco-regional control in advanced head and neck squamous cell carcinoma treated by radiation therapy Radiother Oncol 2000 57 39 43 10.1016/S0167-8140(00)00223-1 11033187

[B67] Nordsmark M Bentzen SM Rudat V Brizel D Lartigau E Stadler P Becker A Adam M Molls M Dunst J Terris DJ Overgaard J Prognostic value of tumor oxygenation in 397 head and neck tumors after primary radiation therapy: An international multi-center study Radiother Oncol 2005 77 18 24 10.1016/j.radonc.2005.06.038 16098619

[B68] Nordsmark M Overgaard J Tumor hypoxia is independent of hemoglobin and prognostic for loco-regional tumor control after primary radiotherapy in advanced head and neck cancer Acta Oncol 2004 43 396 403 10.1080/02841860410026189 15303502

[B69] Lassen P Eriksen JG Krogdahl A Therkildsen MH Ulhoi BP Overgaard M Specht L Andersen E Johansen J Andersen LJ Grau C Overgaard J The influence of HPV-associated p16-expression on accelerated fractionated radiotherapy in head and neck cancer: Evaluation of the randomized DAHANCA 6&7 trial Radiother Oncol 2011 100 49 55 10.1016/j.radonc.2011.02.010 21429609

[B70] Glaser CM Millesi W Kornek GV Lang S Schull B Watzinger F Selzer E Lavey RS Impact of hemoglobin level and use of recombinant erythropoietin on efficacy of preoperative chemoradiation therapy for squamous cell carcinoma of the oral cavity and oropharynx Int J Radiat Oncol Biol Phys 2001 50 705 715 10.1016/S0360-3016(01)01488-2 11395239

[B71] Ang KK Harris J Wheeler R Weber R Rosenthal DI Nguyen-Tan PF Westra WH Chung CH Jordan RC Lu C Kim H Axelrod R Silverman CC Redmond KP Gillison ML Human papillomavirus and survival of patients with oropharyngeal cancer N Engl J Med 2010 363 24 35 10.1056/NEJMoa0912217 20530316 PMC2943767

[B72] Licitra L Perrone F Bossi P Suardi S Mariani L Artusi R Oggionni M Rossini C Cantu G Sguadrelli M Quattrone P Locati LD Bergamini C Olmi P Pierotti MA Pilotti S High-risk human papillomavirus affects prognosis in patients with surgically treated oropharyngeal squamous cell carcinoma J Clin Oncol 2006 24 5630 5636 10.1200/JCO.2005.04.6136 17179101

[B73] Lassen P Eriksen JG Hamilton-Dutoit S Tramm T Alsner J Overgaard J Effect of HPV-associated p16INK4A expression on response to radiotherapy and survival in squamous cell carcinoma of the head and neck J Clin Oncol 2009 27 1992 1998 10.1200/JCO.2008.20.2853 19289615

